# Supplementary material for: Association of CYP24A1 Gene rs6127099 (A > T) Polymorphism with Lower Risk to COVID-19 Infection in Kazakhstan
Source: Genes (Basel). 2023 Jan 24;14(2):307. doi: 10.3390/genes14020307 (PMC9957291; doi:10.3390/genes14020307)
Supplement: Supplementary file 1 [file genes-14-00307-s001.zip › genes-2079761-supplementary.pdf]

Table S1. Association of candidate SNPs with symptomless COVID-19. Genotype frequencies and inheritance patterns of selected SNPs stratified by gender and age categories.

Model	SNP [Gene]	Total			Gender-based stratification				Age-based stratification					
					Male		Female		<40		40-60		60+	
		Case/Control	OR (95% CI)	p-value	OR (95% CI)	p-value	OR (95% CI)	p-value	OR (95% CI)	p-value	OR (95% CI)	p-value	OR (95% CI)	p-value
	**rs731236 TaqI [*VDR*]**													
**Additive**	AA	74/34	Reference		Reference		Reference		Reference		Reference		Reference	
	AG	50/12	1.91 (0.9–4.05)	0.089	1.8 (0.7 - 4.61)	0.221	1.92 (0.54 - 6.86)	0.309	3.03 (0.81-11.31)	0.098	1.28 (0.38-4.27)	0.691	3.11 (0.5-19.54)	0.226
	GG	4/0	1	1.000	1.00		1.00		1.00		1.00		1.00	
**Dominant**	AA	74/34	Reference		Reference		Reference		Reference		Reference		Reference	
	AG+GG	54/12	2.07 (0.98–4.36)	0.056	2.07 (0.59 - 7.3)	0.258	1.95 (0.76 - 4.98)	0.162	3.3 (0.89-12.22)	0.074	1.34 (0.4-4.48)	0.630	3.5 (0.57-21.67)	0.178
**Recessive**	AA+AG	124/46	Reference		Reference		Reference		Reference		Reference		Reference	
	GG	4/0	1	1.000	1	1.000	1	1.000	1.00		1.00		1.00	
**Allelic**	A	198/80	Reference		Reference		Reference		Reference		Reference		Reference	
	G	58/12	1.95 (1.00–3.83)	0.051	2 (0.62-6.42)	0.244	1.83 (0.8-4.21)	0.152	3.1 (0.89-10.76)	0.075	1.3 (0.49-3.49)	0.600	3.08 (0.6-15.88)	0.179
	**rs1544410 BsmI [*VDR*]**													
**Additive**	TT	4/0	Reference		Reference		Reference		Reference		Reference		Reference	
	TC	57/13	2.13 (1.02–4.42)	0.043	1.44 (0.44 - 4.79)	0.544	2.48 (0.97 - 6.34)	0.058	3.66 (0.99-13.56)	0.052	1.03 (0.31-3.43)	0.962	6.42 (1.00-41.21)	0.05
	CC	68/33	1	1.000	1.00		1.00		1.00		1.00		1.00	
**Dominant**	TT	4/0	Reference		Reference		Reference		Reference		Reference		Reference	
	TC+CC	125/46	1	1.000	1.00		1.00		1.00		1.00		1.00	
**Recessive**	TT+TC	61/13	Reference		Reference		Reference		Reference		Reference		Reference	
	CC	68/33	0.44 (0.21–0.91)	0.027	0.64 (0.2 -2.12)	0.469	0.38 (0.15 - 0.96)	0.041	0.25 (0.07-0.94)	0.040	0.93 (0.28-3.07)	0.899	0.14 (0.02-0.91)	0.039
**Allelic**	T	65/13	Reference		Reference		Reference		Reference		Reference		Reference	
	C	193/79	0.49 (0.25–0.94)	0.031	0.64 (0.22-1.9)	0.424	0.45 (0.2-1.02)	0.057	0.28 (0.08-0.98)	0.046	0.89 (0.35-2.31)	0.814	0.22 (0.04-1.12)	0.068
	**rs2228570 FokI [*VDR*]**													
**Additive**	AA	14/4	Reference		Reference		Reference		Reference		Reference		Reference	
	AG	61/17	1.02 (0.3–3.52)	0.968	1.75 (0.32 -9 .47)	0.516	0.5 (0.06 - 4.47)	0.535	0.49 (0.05-4.43)	0.522	0.63 (0.06-6.8)	0.700	1.5 (0.25-8.98)	0.657

	GG	56/25	0.64 (0.19–2.14)	0.469	1.97 (0.36 - 10.82)	0.436	0.21 (0.02 - 1.74)	0.147	0.21 (0.02-1.78)	0.152	0.66 (0.06-6.8)	0.724	1.00	
**Dominant**	AA	14/4	Reference		Reference		Reference		Reference		Reference		Reference	
	AG+GG	117/42	0.8 (0.25–2.55)	0.701	1.85 (0.37 - 9.17)	0.76	0.3 (0.04 - 2.46)	0.262	0.3 (0.04-2.48)	0.264	0.64 (0.07-6.26)	0.704	1.00	
**Recessive**	AA+AG	75/21	Reference		Reference		Reference		Reference		Reference		Reference	
	GG	56/25	0.63 (0.32–1.23)	0.176	1.26 (0.43 - 3.66)	0.671	0.37 (0.15 - 0.93)	0.034	0.38 (0.14-1.01)	0.053	0.97 (0.29-3.18)	0.956	1 (0.18-5.46)	1.00
**Allelic**	A	89/25	Reference		Reference		Reference		Reference		Reference		Reference	
	G	173/67	0.73 (0.43–1.23)	0.231	1.3 (0.6-2.83)	0.504	0.44 (0.2-0.93)	0.032	0.44 (0.2-0.98)	0.045	0.9 (0.35-2.31)	0.829	1.6 (0.5-5.1)	0.427
	**rs6013897 [*CYP24A1*]**													
**Additive**	AA	3/1	Reference		Reference		Reference		Reference		Reference		Reference	
	AT	34/13	0.87 (0.08–9.15)	0.909	0.73 (0.23-2.37)	0.604	1.64 (0.13-20.94)	0.702	0.79 (0.29-2.14)	0.638	0.4 (0.09-1.78)	0.231	4.45 (0.45-44.4)	0.203
	TT	94/32	0.98 (0.1–9.75)	0.986	1.00		1.64 (0.14-19.14)	0.694	1.00		1.00		1.00	
**Dominant**	AA	3/1	Reference		Reference		Reference		Reference		Reference		Reference	
	AT+TT	128/45	0.95 (0.1–9.35)	0.964	1.00		1.64 (0.14-18.85)	0.691	1.00		1.00		1.00	
**Recessive**	AA+AT	37/14	Reference		Reference		Reference		Reference		Reference		Reference	
	TT	94/32	1.11(0.53–2.32)	0.778	1.25 (0.39-3.99)	0.706	1.05 (0.4-2.73)	0.922	1.17 (0.43-3.16)	0.762	2.06 (0.49-8.66)	0.324	0.45 (0.07-2.81)	0.392
**Allelic**	A	40/15	Reference		Reference		Reference		Reference		Reference		Reference	
	T	222/77	1.08 (0.57–2.07)	0.813	1.1 (0.39-3.13)	0.859	1.09 (0.48-2.5)	0.833	1.03 (0.43-2.46)	0.06	1.6 (0.43-5.93)	0.479	0.83 (0.19-3.67)	0.805
	**rs6127099 [*CYP24A1*]**													
**Additive**	AA	68/13	Reference		Reference		Reference		Reference		Reference		Reference	
	AT	49/25	0.37 (0.17–0.8)	0.012	0.24 (0.07 - 0.8)	0.02	0.46 (0.17-1.27)	0.135	0.17 (0.05-0.57)	0.004*	0.42 (0.1-1.72)	0.229	1.88 (0.34-10.46)	0.474
	TT	8/7	0.22 (0.07–0.71)	0.011	0.07 (0.01-0.78)	0.031	0.31 (0.072-1.36)	0.122	0.24 (0.04-1.69)	0.153	0.11 (0.01-0.79)	0.028	0.63 (0.03-12.4)	0.758
**Dominant**	AA	68/13	Reference		Reference		Reference		Reference		Reference		Reference	
	AT+TT	57/32	0.34 (0.16–0.71)	0.004*	0.21 (0.07-0.65)	0.007	0.43(0.16-1.14)	0.089	0.18 (0.05-0.58)	0.004*	0.31 (0.08-1.15)	0.08	1.56 (0.31-1.82)	0.587
**Recessive**	AA+AT	117/38	Reference		Reference		Reference		Reference		Reference		Reference	
	TT	8/7	0.37 (0.13–1.09)	0.072	0.13 (0.01-1.39)	0.092	0.49 (0.13-1.83)	0.290	0.71 (0.13-3.95)	0.695	0.17 (0.03-1.04)	0.055	0.47 (0.03-8.52)	0.610

**Allelic**	A	185/51	Reference		Reference		Reference		Reference		Reference		Reference	
	T	65/39	0.46 (0.28–0.76)	0.002*	0.29 (0.12-0.66)	0.003*	0.56 (0.29-1.07)	0.079	0.4 (0.2-0.82)	0.013	0.35 (0.14-0.85)	0.02	1.14 (0.33-4.00)	0.833
	**rs7041 [*GC*]**													
**Additive**	AA	52/15	Reference		Reference		Reference		Reference		Reference		Reference	
	AC	65/21	0.89 (0.42–1.9)	0.769	0.81 (0.24-2.72)	0.739	0.95 (0.36-2.51)	0.915	2.01 (0.65-6.2)	0.224	0.42 (0.1-1.8)	0.241	0.48 (0.07-3.09)	0.437
	CC	12/10	0.34 (0.13–0.96)	0.041	0.27 (0.05-1.33)	0.107	0.42 (0.11-1.61)	0.208	0.52 (0.15-1.82)	0.305	0.13 (0.01-1.1)	0.061	0.29 (0.01-6.91)	0.441
**Dominant**	AA	52/15	Reference		Reference		Reference		Reference		Reference		Reference	
	AC+CC	77/31	0.72 (0.35–1.46)	0.357	0.62 (0.2-1.92)	0.405	0.79 (0.32-1.99)	0.622	1.26 (0.48-3.31)	0.632	0.34 (0.08-1.42)	0.140	0.45 (0.07-2.81)	0.392
**Recessive**	AA+AC	117/36	Reference		Reference		Reference		Reference		Reference		Reference	
	CC	12/10	0.37 (0.15–0.93)	0.034	0.3 (0.07-1.27)	0.102	0.44 (0.13-1.47)	0.182	0.38 (0.12-1.2)	0.100	0.22 (0.03-1.49)	0.122	0.47 (0.03-8.52)	0.610
**Allelic**	A	169/51	Reference		Reference		Reference		Reference		Reference		Reference	
	C	89/41	0.66 (0.4–1.06)	0.087	0.57 (0.27-1.22)	0.150	0.72 (0.38-1.37)	0.320	0.82 (0.41-1.64)	0.580	0.46 (0.19-1.09)	0.079	0.63 (0.2-1.99)	0.427
	**rs4588 [*GC*]**													
**Additive**	GG	58/26	Reference		Reference		Reference		Reference		Reference		Reference	
	GT	55/16	1.54 (0.75–3.18)	0.242	1.71 (0.55-5.3)	0.349	1.44 (0.55-3.72)	0.456	2.1 (0.71-6.24)	0.183	0.84 (0.24-2.9)	0.786	2.25 (0.42-12.09)	0.345
	TT	8/1	3.59 (0.43–30.17)	0.240	1.00		1.44(0.15-13.97)	0.755	1.68 (0.17-16.48)	0.657	1.00		1.00	
**Dominant**	GG	58/26	Reference		Reference		Reference		Reference		Reference		Reference	
	GT+TT	63/17	1.66 (0.82–3.37)	0.160	2.08 (0.68-6.31)	0.198	1.44 (0.57-3.62)	0.443	2.04 (0.72-5.76)	0.18	1 (0.29-3.41)	1.00	2.5 (0.47-13.27)	0.282
**Recessive**	GG+GT	113/42	Reference		Reference		Reference		Reference		Reference		Reference	
	TT	8/1	2.97 (0.36–24.5)	0.311	1.00		1.23 (0.13-11.53)	0.858	1.25 (0.13-11.83)	0.848	1.00		1.00	
**Allelic**	G	171/68	Reference		Reference		Reference		Reference		Reference		Reference	
	T	71/18	1.57 (0.87–2.83)	0.134	2.1 (0.82-5.37)	0.122	1.3 (0.61-2.77)	0.500	1.65 (0.7-3.91)	0.254	1.22 (0.45-3.28)	0.691	2.2 (0.53-9.18)	0.279
	**rs10877012 [*CYP27B1*]**													
**Additive**	GG	15/11	Reference		Reference		Reference		Reference		Reference		Reference	
	GT	77/24	2.35 (0.95–5.8)	0.063	16.9 (1.83 -156.6)	0.013	1.26 (0.38 -4.16)	0.7	1.22 (0.33-4.48)	0.762	2.44 (0.41-14.47)	0.325	1.05 (0.14-7.93)	0.964
	TT	36/9	2.93 (1.01–8.53)	0.048	36 (2.69 -481.21)	0.007	1.22 (0.33-4.6)	0.764	1.42(0.29-6.81)	0.664	4 (0.52-30.76)	0.183	1.00	
**Dominant**	GG	15/11	Reference		Reference		Reference		Reference		Reference		Reference	

	GT+TT	113/33	2.51 (1.05–5.99)	0.038	19.85 (2.19 -180.2)	0.008	1.25 (0.4 - 3.89)	0.700	1.27 (0.36-4.47)	0.709	2.83 (0.5-15.99)	0.238	1.00	
**Recessive**	GG+GT	92/35	Reference		Reference		Reference		Reference		Reference		Reference	
	TT	36/9	1.52 (0.67–3.48)	0.320	3.19 (0.64-15.92)	0.158	1.03 (0.38 - 2.78)	0.956	1.21 (0.36-4.12)	0.756	1.92 (0.45-8.11)	0.375	2.23 (0.36-14.00)	0.392
**Allelic**	G	107/46	Reference		Reference		Reference		Reference		Reference		Reference	
	T	149/42	1.53 (0.94–2.48)	0.089	2.56 (1.17-5.58)	0.018	1.08 (0.57-2.04)	0.807	1.15 (0.57-2.31)	0.701	1.64 (0.7-3.87)	0.256	3.57 (1.09-11.66)	0.035
	**rs1074165 [*CYP2R1*]**													
**Additive**	GG	89/30	Reference		Reference		Reference		Reference		Reference		Reference	
	GA	34/15	0.76 (0.37–1.59)	0.473	2.07 (0.58-7.4)	0.261	0.41 (0.16-1.05)	0.063	0.77 (0.27-2.2)	0.627	1.31 (0.3-5.68)	0.719	0.44 (0.08-2.27)	0.324
	AA	2/0	1	1.000	1.00		1.00		1.00		-		-	
**Dominant**	GG	89/30	Reference		Reference		Reference		Reference		Reference		Reference	
	GA+AA	36/15	0.81 (0.39–1.68)	0.570	2.22 (0.63-7.87)	0.216	0.43 (0.17-1.09)	0.077	0.86 (0.3-2.42)	0.771	1.31 (0.3-5.68)	0.719	0.44 (0.08-2.27)	0.324
**Recessive**	GG+GA	123/45	Reference		Reference		Reference		Reference		Reference		Reference	
	AA	2/0	1	1.000	1.00		1.00		1.00		1.00		1.00	
**Allelic**	G	212/75	Reference		Reference		Reference		Reference		Reference		Reference	
	A	38/15	0.9 (0.47–1.72)	0.742	2.12 (0.66-6.81)	0.208	0.54 (0.24-1.21)	0.136	0.99 (0.39-2.49)	0.975	1.26 (0.32-4.97)	0.739	0.56 (0.14-2.16)	0.398
	**rs1747027 [*CYP27A1*]**													
**Additive**	AA	70/18	Reference		Reference		Reference		Reference		Reference		Reference	
	AT	34/16	0.55 (0.25–1.2)	0.133	0.21 (0.06-0.72)	0.013	1.08 (0.35-3.34)	0.889	1.09 (0.34-3.48)	0.883	0.24 (0.05-1.1)	0.067	0.27 (0.05-1.51)	0.135
	TT	9/2	1.16 (0.23–5.83)	0.860	1.00		0.88 (0.16-4.87)	0.879	1.64 (0.17-15.4)	0.667	0.6 (0.05-7.01)	0.684	-	
**Dominant**	AA	70/18	Reference		Reference		Reference		Reference		Reference		Reference	
	AT+TT	43/18	0.61 (0.29–1.31)	0.206	0.27 (0.08-0.86)	0.027	1.03 (0.37-2.91)	0.954	1.17 (0.39-3.53)	0.782	0.3 (0.07-1.23)	0.094	0.27(0.05-1.51)	0.135
**Recessive**	AA+AT	104/34	Reference		Reference		Reference		Reference		Reference		Reference	
	TT	9/2	1.47 (0.3–7.14)	0.632	1.00		0.85 (0.16-4.48)	0.847	1.58 (0.18-14.13)	0.683	0.97 (0.09-10.38)	0.978	1.00	
**Allelic**	A	174/52	Reference		Reference		Reference		Reference		Reference		Reference	
	T	52/20	0.78 (0.43–1.42)	0.411	0.46 (0.18-1.19)	0.109	0.98 (0.43-2.22)	0.963	1.2 (0.49-2.92)	0.688	0.46 (0.15-1.37)	0.163	0.37 (0.09-1.62)	0.187
	**rs1800629 [*TNF–α*]**													
**Additive**	AA	2/2	Reference		Reference		Reference		Reference		Reference		Reference	

	AG	30/9	3.33 (0.41–27.13)	0.260	1.55 (0.43-5.58)	0.504	7.2 (0.54-96.6)	0.136	-		2 (0.2-19.9)	0.554	1.00
	GG	95/35	2.71 (0.37–20.01)	0.327	1.00		6.74(0.58-78.42)	0.128	0.95 (0.31-2.96)	0.932	3.11 (0.38-25.38)	0.289	1.00
**Dominant**	AA	2/2	Reference		Reference		Reference		Reference		Reference		Reference
	AG+GG	125/44	2.84 (0.39–20.78)	0.304	1.00		6.83 (0.59-78.65)	0.123	1.00		2.77 (0.35-21.73)	0.332	1.00
**Recessive**	AA+AG	32/11	Reference		Reference		Reference		Reference		Reference		Reference
	GG	95/35	0.93 (0.42–2.05)	0.863	0.6 (0.17-2.13)	0.426	1.24(0.45-3.4)	0.674	0.95 (0.31-2.96)	0.932	1.87 (0.53-6.58)	0.332	1.00
**Allelic**	A	34/13	Reference		Reference		Reference		Reference		Reference		Reference
	G	220/79	1.06 (0.53–2.12)	0.858	0.59 (0.18-1.91)	0.377	1.53 (0.65-3.6)	0.332	0.96 (0.33-2.76)	0.936	1.94 (0.7-5.36)	0.202	1.00
